# Knowledge and practice to prevent COVID-19 and its associated factors among pregnant women in Debre Tabor Town Northwest Ethiopia, a community-based cross-sectional study

**DOI:** 10.1186/s12884-021-03877-4

**Published:** 2021-05-21

**Authors:** Alemu Degu Ayele, Gedefaye Nibret Mihretie, Habtamu Gebrehana Belay, Adanech Getie Teffera, Bekalu Getnet Kassa, Bedemariam Tadesse Amsalu

**Affiliations:** College of Health Sciences, Debre Tabor University, Debre Tabor, Ethiopia

**Keywords:** COVID-19, Knowledge, Practice, Pregnant women, Debre Tabor

## Abstract

**Background:**

Coronavirus disease is now a global concern with the non-availability of antiviral treatment and attacks all groups of the population. Hence, applying preventive measures is the most critical intervention to control the infection. Pregnant women are particularly susceptible to respiratory pathogens because of their immunosuppressive state and physiological adaptive change during pregnancy. Therefore, this study was aimed to assess knowledge and practice to prevent coronavirus disease and its associated factors among pregnant women in Debre Tabor Town.

**Methods:**

Community-based cross-sectional study was conducted among 422 participants from May 25–June 15, 2020. A simple random sampling technique was employed. Data were collected by face-to-face interview using a structured and pre-tested questionnaire and analysis using SPSS version 23. Bivariable and multivariable logistic regression analysis was carried out and *p*-value < 0.05 at 95% CI were considered as statistically significant.

**Results:**

Overall 46.8 and 47.6% of women were knowledgeable and had good practice to prevent coronavirus respectively. Women’s age (15–24 years) (AOR = 4.85, 95% CI: 1.34–5.42), educational status (AOR:3.70; 95% CI: 1.16–5.40) being civil servant (AOR:2.84; 95% CI: 1.55–5.21), wanted pregnancy (AOR:3.37; 95% CI: 1.20–9.45), antenatal care follow-up (AOR:2.07; 95% CI: 1.03–4.13) were significantly associated with COVID-19 knowledge, whereas educational status (AOR:3.78; 95% CI: 1.19–5.11), number of children (AOR:2.89; 95% CI: 1.29–6.45) and knowledge (AOR:8.42;95% CI: 4.50–15.85), were also found to be statistically significant with practice.

**Conclusions:**

Most of the participants had poor knowledge and inappropriate practice. Increasing health education programs via different media, coordinated and combined efforts of authorities and all individuals will be needed to battles the spread of the infection.

**Supplementary Information:**

The online version contains supplementary material available at 10.1186/s12884-021-03877-4.

## Background

COVID-19 is an emerging respiratory disease caused by a single-stranded novel coronavirus. It is highly infectious and characterized by different signs and symptoms such as fever, dry cough, fatigue, myalgia, and dyspnea [1–4].

The first human case of COVID-19 was reported in Wuhan City, China, in December 2019. The World Health Organization (WHO) declared the 2019–20 COVID-19 outbreaks as a Public Health Emergency of International Concern (PHEIC) on 30 January 2020 and a pandemic on 11 March 2020 [1, 5].

COVID-19 can be transmitted through the respiratory droplet, physical contact, feco-oral, and has an incubation period of 2–14 days. The infected persons can also transmit the virus via close contact and respiratory droplets before they become symptomatic [6–8]. Nowadays the COVID-19 pandemic is causing huge stress both socially and economically on the health care system of the globe [9].

According to European Centre for Disease Prevention and Control report from 31 December 2019 to 28 June 2020, there were a total of 9, 952, 507 COVID-19 cases and 498,519 deaths globally. Among these, 371,448 cases and 9480 deaths were from Africa [10]. Pregnant women are particularly susceptible to respiratory pathogens and severe pneumonia, because of their immunosuppressive state, and physiological adaptive changes during pregnancy [11]. Pregnancy is also a state of partial immune suppression which makes them more vulnerable to viral infections [12].

Even though there is no clear evidence to support the vertical transmission of the COVID-19, studies support that maternal deaths are evident as a consequence of the pandemic. Particularly, from February 26, 2020, until June 18, 2020, Brazil reported 124 maternal deaths [13]. Various studies also noticed that pregnant women with confirmed COVID-19 had pre-existing medical comorbidities including diabetic Mellitus, bacterial and viral co-infections, and obstetric complications including pre-eclampsia, cesarean delivery, preterm birth, premature rupture of membrane, placenta previa, and post-partum hemorrhage. Besides, adverse birth outcomes such as fetal distress, fetal tachycardia, low birth weight, neonatal asphyxia, and stillbirth were strongly associated with COVID-19 [14–16]. Therefore, pregnant women and neonates require special attention in the prevention, diagnosis, and management of COVID-19.

Ethiopia is one of the African countries expected to be most severely hit by the global COVID-19 pandemic due to limited trained healthcare professionals and material resources [17]. The government of Ethiopia implements various actions ranging from public health emergency response to lockdown. The policy segmented the population by tailoring activities of risk communication and community engagement at all levels [18].

Even though the government of Ethiopia has been motivated and showing a high commitment to control the pandemic, the spread of the disease was increased each day. Notably, in Ethiopia, as of June 28, 2020, there were a total of 5689 cases, of which 98 deaths, 33 critical cases, 3459 active cases, and 2132 recovered cases reported by the Ministry of Health and the Ethiopian Public Health Institute [19].

Despite, currently the COVID-19 vaccine is available to prevent the infection, there is no effective antiviral drug for the definitive treatment of the COVID-19 pandemic. So, increasing the level of knowledge and applying preventive measures to control COVID-19 infection is the most critical intervention. Therefore, this study was aimed to investigate knowledge and practice to prevent the infection and its associated factor among pregnant women in Debre Tabor Town Northwest Ethiopia.

## Methods

### Study design and setting

A community-based cross-sectional study design was conducted from May 25–June 15, 2020 in Debre Tabor Town.

### Study setting

Debre Tabor Town is the capital city of South Gondar Zone, Amhara region, Northwest Ethiopia. The town is located 665 km northwest of Addis Ababa (the capital city of Ethiopia). The town has six small administrative units called kebeles with a total population of 85,727 of whom 49.6% (42,521) and 50.4% (43,206) were men and women respectively and 19,936 households (HH) based on 2020 information obtained from the town health office [20].

Among women, 20.96% (17,968) were of reproductive age and 1230 pregnant women were residing in the town during the study period.

### Participants

The study populations were all pregnant women in Debre Tabor Town. Pregnant women who gave informed consent to participate in the study and lived in the town for at least 6 months during the data collection period were included.

### Variables

Knowledge and practice to prevent COVID-19 were the outcomes of interests and independent variables were socio-demographic characteristics (age, religion, educational status, occupational status, husband educational, occupational status), Reproductive characteristics (gravidity, parity, number of children, history of abortion, ANC follow-up and condition of pregnancy), knowledge-related as well as practice-related factors.

### Operational definitions

Level of knowledge and practice were determined using 15 knowledge assessing and 6 practice assessing questionnaires respectively. A value of 1 and 0 was given for each correct and incorrect answer respectively and labeled as good and poor knowledge and practice based on mean score.

- **Good knowledge:** Participants who scored greater than or equal to the mean score.
- **Poor knowledge:** Participants who score less than the mean score.
- **Good practice:** Participants who scored greater than or equal to the mean score.
- **Poor practice:** Participants who scored less than the mean score.

### Data collection tool and quality management

Data were collected via face-to-face interview techniques using a structured and pre-tested questionnaire by applying all the possible strict preventive measures of the pandemic. The questionnaire was first prepared in English, then translated to the local language, Amharic, for simplicity, and back to English for consistency, by two different language expert individuals who speak both English and Amharic fluently. The questionnaire was adapted from WHO guidelines and related kinds of literature in different parts of the world and modified according to the local context. The questionaries have four items (socio-demographic characteristics, reproductive health-related characteristics, knowledge-related characteristics, and practice-related characteristics). Pre-testing of the questionnaire was done on 10% of the total participants (42 pregnant women) in Woreta Town near to the study setting. During the pre-test, the questionnaire was assessed for its clarity, accuracy, comprehensiveness, readability, and optimal time for completing the interview. Modifications and corrections including wording, logical sequence and skip patterns were immediately performed based on the results. The data were collected by six diploma health professionals and supervised by two BSc health professionals. Data collectors and supervisors were trained for 1 day on the aim of the study, method of data collection, contents of the questionnaire, confidentiality, and informed consent before they start the actual data collection. The completeness and consistency of the collected data were cross-cheeked, cleaned, and compiled by supervisors and principal investigator regularly.

### Sample size determination and sampling procedure

A sample size of 422 participants was determined by using the single proportion formula with the following assumptions: the proportion of knowledge and practice of preventive measures against COVID-19 is 50% since there is no related study in Ethiopia, confidence interval of (CI) 95%, margin of error (d) 5%, and considering none response rate of 10%.

A simple random sampling technique was employed to select 422 pregnant women of 1230 pregnant women residing in the town. Households were sampling units and samples were selected and proportionally allocated to each kebeles based on their total numbers of households. The number of pregnant women with their household number was got from the family folder of health extension workers (HEWs). Then the study households were selected from each kebeles through a simple random sampling technique by using a table of random numbers starting from kebele one from a random start point. One pregnant woman per household was interviewed. When two or more eligible pregnant women were found in one household, only one was interviewed by using the lottery method and if no eligible women were identified in the selected household, the next eligible household located in the clockwise direction was visited and included until we got the desired sample size.

### Data processing, analysis, and interpretation

The collected data were encoded and entered into Epi-Data version 4.2 and then exported to SPSS version 23 for analysis. Descriptive analysis was carried out and frequency tables and percentages were used to present the descriptive results.

Bivariable logistic regression analysis was done to examine the crude association dependent and independent variables by computing odds ratio (OR) with 95% CI and then variables with *p*-value ≤0.2 were entered into multivariable logistic regression model for further analysis by controlling confounding factors to see the effect of predictors on the outcome variables and finally, a significant association was declared based on *p* < 0.05 and adjusted odds ratio (AOR) with 95% CI.

### Ethical consideration

Ethical clearance was obtained from the institutional Ethics Review Committee of Debre Tabor University. Written informed consent was obtained from each study participant. Confidentiality was assured by excluding any personal identifiers during the study.

## Results

### Socio-demographic characteristics

A total of 405 pregnant women have participated in this study with a response rate of 95.9%. Among these participants more than half of the women 230 (56.8%) were belonged to the age group of 25–34 years ranging from 20 to38 years. The mean age of the participant was 27.15 (SD ± 4.719) years. The study participants were predominantly Amhara 396(97.8%) and Orthodox Christian followers 370 (91.4%). Of the total respondents, almost half of the respondents 202 (49.9%) and 215(54%) of participants husband had college and above education (Table 1).

**Table 1:** Socio -demographic characteristics of pregnant women in Debre Tabor Town Northwest Ethiopia, May 25–June 15, 2020(*N* = 405)

Variables	Frequency	Percent (%)
Age in years		
15–24	127	31.4
25–34	230	56.8
≥ 35	48	11.9
Ethnicity		
Amhara	396	97.8
Others *	9	2.2
Religion		
Orthodox	370	91.4
Muslim	28	6.9
Others**	7	1.7
Educational status		
No formal education	60	14.8
Primary	71	17.5
Secondary	72	17.8
College and above	202	49.9
Occupation		
Housewife	173	42.7
Civil servant	115	28.4
Private business	102	25.2
Others***	15	3.8
Marital status		
Married	398	98.3
Others ****	7	1.7
Husband educational status		
No formal education	43	10.8
Primary	36	9.0
Secondary	104	26.2
College and above	215	54.0
Husband occupation(*N* = 398)		
Civil servant	219	55.0
Private business	136	34.2
Employed at private sector	29	7.3
Daily laborer	14	3.5

*Oromo, Tigray, Gurage ** protestant, catholic *** student, job finder, ****widowed, divorced

### Obstetric and reproductive health characteristics

This study revealed that about 253(62.5%) and 162(40%) of participants were multigravidas and multiparas respectively. Concerning the condition of pregnancy 330 (81.5%) of their current pregnancies were wanted and planned. More than three-fourth of the participants 324(80.0%) had antenatal care (ANC) follow-up in their current pregnancies. On the subject of the number of children, 285 (70.4%) women had less than or equal to three alive children (Table 2).

**Table 2:** Obstetrics and reproductive characteristics of pregnant women in Debre Tabor Town, Northwest Ethiopia, May 25–June 15, 2020(*N* = 405)

Variables	Frequency	Percent (%)
Gravidity		
Primi	152	37.5
Multi	253	62.5
Parity		
Nulliparous	160	39.5
Primipara	83	20.5
Multipara	162	40.0
ANC follow-up		
Yes	324	80.0
No	81	20.0
No of ANC visit (*N* = 324)		
> 3	167	51.5
≤ 3	157	48.5
Number of living children		
≤ 3 children	285	70.4
> 3 children	120	29.6
Condition of pregnancy		
Unwanted	33	8.1
Wanted	330	81.5
Mistimed	42	10.4
Previous adverse pregnancy outcomes		
Yes	76	18.8
No	329	81.2
Types of adverse pregnancy outcomes(*N* = 76)		
Abortion	28	36.8
Preterm labor	12	15.8
Low birth weights	23	30.2
Stillbirths	13	17.2
Chronic health problem		
Yes	31	7.7
No	374	92.3
Types of chronic health problem(*N* = 31)		
HIV/AIDIS	6	19.4
Hypertension	8	25.8
Renal stone	10	32.2
Others^a^	7	22.6

^a^other chronic health problems: anemia, diabetic mellitus and tuberculosis

### Knowledge of study participants about COVID-19

**Fig. 1:**
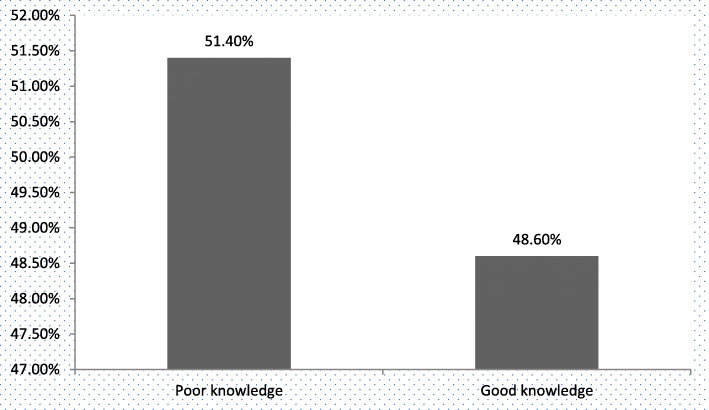
Knowledge of pregnant women about COVID-19; Debre Tabor Town Northwest Ethiopia, May 25–June 15, 2020(*N* = 405)

According to the finding of this study, the entire participants 405 (100%) have been heard about COVID-19. Mass media 308(76.0%), health workers 95(23.5%), and social media 49 (12.1%) were the main sources of information. Fever 296(73.1%) and dry cough 233(57.5% were the two most common symptoms of COVID-19 mentioned by the participants (Table 3). According to this study, the mean score of knowledge was 7.600 (SD ± 2.216), and 197 (48.6%) with 95% CI (43.5–53.8) of the participants had adequate knowledge about COVID-19 (Fig. 1).

**Table 3:** Knowledge of pregnant women in Debre Tabor Town, Northwest Ethiopia, May 25–June 15, 2020(*N* = 405)

Knowledge questions	Frequency	Percent (%)
Ever heard about COVID-19		
Yes	405	100
No	0	0
COVID-19 is viral disease		
Yes	261	64.4
No	144	35.6
Respiratory droplets and close contact are the main transmission route		
Yes	357	88.1
No	48	11.9
Incubation period of COVID-19 is 2–14 days		
Yes	183	45.2
No	222	54.8
All peoples are generally susceptible for COVID-19		
Yes	200	49.4
No	105	50.6
Fever is a symptom of COVID-19		
Yes	296	73.1
No	109	26.9
Dry cough is a symptom of COVID-19		
Yes	233	57.5
No	172	42.5
Headache is a symptom of COVID-19		20.7
Yes	82	
No	321	79.3
Sore throat is a symptom of COVID-19		
Yes	68	16.8
No	337	83.2
Runny nose is a symptom of COVID-19		
Yes	95	23.5
No	310	76.5
Difficulty of breathing is a symptom of COVID-19		
Yes	91	22.5
No	314	77.5
Stay at home and wearing face mask can prevent transmission of COVID-19		
Yes	178	44.0
No	227	56.0
People with co-existing disease and smokers had poor prognostic outcomes if infected with COVID.		
Yes	198	48.9
No	207	51.1
Person with COVID-19 can transmit the virus to others without development of signs and symptoms.		
Yes	262	64.7
No	143	35.3
Pregnant women are at high risk than others if infected with COVID −19		
Yes	167	41.2
No	238	58.8

### Practice of study participant to prevent COVID-19

The majority of the participants 309(76.3%) have been practiced at least one preventive measure against COVID-19 while 96(23.7%) did not apply any type of preventive measures to prevent COVID-19 infection. Washing hands with water and soap 271 (87.7%) and cover mouth and nose during coughing or sneezing 238(77%) were the most common practiced preventive measures to combat the spread of the infection (Table 4).

**Table 4:** Practice of pregnant women to prevent against COVID-19 in Debre Tabor Town, Northwest Ethiopia, May 25–June 15, 2020(*N* = 309)

Practice questions	Frequency	Percent (%)
Wash hand with water and soap		
Yes	271	87.7
No	38	12.3
Avoid touching eyes and mouth with unwashed hand.		
Yes	205	66.3
No	104	33.7
Cover mouth and nose during coughing or sneezing		
Yes	238	77.0
No	71	23.0
Wear face mask in public		
Yes	238	77.0
No	71	23.0
Stay at home or in door		
Yes	200	64.7
No	109	35.3
Maintain 1 m distance from others		
Yes	182	58.9
No	127	41.1

**Fig. 2:**
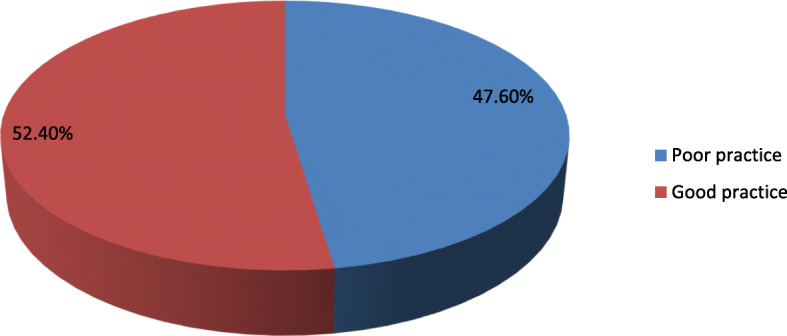
Practice of pregnant women to prevent COVID-19; Debre Tabor Town Northwest Ethiopia, May 25–June 15, 2020(*N* = 405)

According to this study, the mean score of practice was 3.776 (SD ± 1.418), and only 147(47.6%) with 95% CI of (42.1–54.4) participants had a good level of practice to prevent COVID-19 (Fig. 2).

**Fig. 3:**
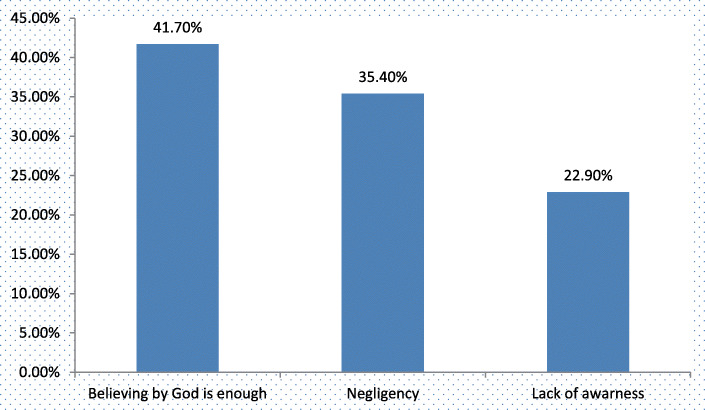
Reasons of pregnant women for not practicing preventive measures against COVID-19; Debre Tabor Town Northwest Ethiopia, May 25–June 15, 2020(*N* = 96)

In this study, 96 (23.7%) of participants have been never practiced any form of preventive measures to prevent the propagation of COVID-19. Among these believing by God is enough 40 (41.7%) was the major reason followed by negligence 34 (35.4%) (Fig. 3).

### Predictors of participant’s knowledge about COVID-19

In binary logistic regression analysis, age, level of education, occupation, husband education, husband occupation, parity, condition of the current pregnancy, and having ANC follow-up in current pregnancy were significantly associated with knowledge of COVID-19. Through multivariable logistic regression analysis after adjusting other co-variables by using backward likelihood stepwise method; age, level of education, occupation, condition of current pregnancy, and having ANC follow-up were found to be statistically significant with knowledge of COVID-19.

Participants whose age group was found between 15 and 24 years were nearly five times more likely to have a sound knowledge about COVID-19 (AOR: 4.85; 95% CI: 1.34–5.42) as compared to participants whose age were greater than or equal to 35 years. Pregnant women who had attended college and above were nearly four times higher the odds of having an adequate level of COVID-19 knowledge (AOR: 3.70; 95% CI: 1.16–5.40) as compared to those who did not attend formal education.

Employed women were nearly three times higher in the odds of having a good level of COVID-19 knowledge (AOR:2.84; 95% CI: 1.55–5.21) compared to housewives. Similarly, the odds of having good COVID-19 knowledge were nearly two times higher among pregnant women who had a private business (AOR:1.73; 95% CI: 1.06–2.84) as compared to housewives.

Moreover, pregnant women whose pregnancy was wanted were three times more likely to have a sound level of COVID-19 knowledge (AOR: 3.37; 95% CI: 1.20–9.45) as compared to those whose pregnancies were unwanted. Pregnant women who had ANC follow-up in their current pregnancy were two times higher the odds of having a good level of COVID-19 knowledge (AOR: 2.07; 95% CI: 1.03–4.13) compared to their counterparts (Table 5).

**Table 5:** Bivariable and multivariable analysis of factors affecting pregnant women level of knowledge about COVID-19 infection in Debre Tabor Town Northwest Ethiopia, May 25–June 15, 2020(*N* = 405)

Variables	Knowledge of COVID-19		COR (95%CI)	AOR (95%CI)	*P-value*
	Good N (%)	Poor N (%)			
Age in years					
15–24	73 (57.5)	54 (42.5)	**2.704 (1.349–5.421)**	**4.852 (1.721–13.682)**	0.003*
25–34	108 (47)	122 (53)	1.77 (0.921–3.404)	1.663 (0.704–3.928)	0.246
≥ 35	16 (33.3)	32 (66.7)	1	1	
Educational status					
No formal education	17 (28.3)	43 (71.7)	1	1	
Primary	33 (46.5)	38 (53.5)	2.197 (1.059–4.558)	1.188 (0.493–2.86)	0.701
Secondary	27 (37.5)	45 (62.5)	1.518 (0.726–3.171)	0.844 (0.35–2.033)	0.705
College and above	120 (59.4)	82 (40.6)	**3.702 (1.976–6.935)**	**2.507 (1.162–5.407)**	0.019*
Occupation					
Housewife	68 (39.3)	105 (60.7)	1	1	
Civil servant	69 (60)	46 (40)	**2.318 (1.43–3.75)**	**2.848 (1.554–5.21)**	0.001*
Private business	54 (52.9)	48 (47.1)	**1.737 (1.06–2.848)**	**2.883 (1.506–5.517)**	0.001*
Others	6 (40.0)	9 (60.0)	0.662 (0.165–2.648	0.527 (0.108–2.567)	0.428
Husband education					
No formal education	8 (18.6)	35 (81.4)	1	1	
Primary	15 (41.7)	21 (58.3)	**3.125 (1.133–8.618)**	0.85 (0.243–2.976)	0.80
Secondary	39 (37.5)	65 (62.5)	**2.625 (1.106–6.232)**	1.525 (0.529–4.397)	0.435
College and above	129 (60)	86 (40)	**6.562 (2.905–14.82)**	2.705 (0.989–7.398)	0.053
Husband occupation					
Civil servant	122 (55.7)	97 (44.3)	3.144 (0.957–10.33)	1.036 (0.2–5.35)	0.966
Private business	53 (39)	83 (61)	1.596 (0.476–5.352)	0.354 (0.066–1.889)	0.224
Worked at private sector	12 (41.4)	17 (58.6)	**1.765 (0.446–6.979)**	0.496 (0.8–3.077)	0.451
Daily laborer	4 (28.6)	10 (71.4)	1	1	
Gravidity					
Primi	76 (50)	76 (50)	1	1	
Multi	121 (47.8)	132 (52.2)	0.971 (0.613–1.371)	**2.759 (1.394–5.461)**	0.004*
Parity					
Nulli	86 (53.)	74 (46.3)	1	1	
Primi	47 (56.6)	36 (43.4)	1.123 (0.659–1.916)	0.683 (0.275–1.697)	0.412
Multi	64 (39.5)	98 (60.5)	**0.562 (0.361–0.875)**	0.616 (0.295–1.286)	0.197
Condition of px					
Unwanted	9 (27.3)	24 (72.7)	1	1	
Wanted	183 (55.5)	147 (44.5)	**3.32 (1.497–7.361)**	**3.372 (1.202–9.456)**	0.021*
Mistimed	5 (11.9)	37 (88.1)	0.36 (0.108–1.206)	0.203 (0.044–2.932)	0.089
ANC in current px					
Yes	176 (54.3)	148 (45.7%)	**3.398 (1.974–5.847)**	**2.073 (1.039–4.134)**	0.039*
No	21 (25.9)	60 (74.1)	1	1	

*COR* Crude Odds Ratio, *AOR* Adjusted Odds Ratio

* = *P-value* < 0.05 considered as statistically significant

### Predictors of participant’s practice to prevent COVID-19

The result of binary logistic regression analysis showed that educational status, husband’s educational and occupational status, number of alive children, having ANC follow-up in the current pregnancy, and knowledge of COVID-19 were significantly associated with the practice of COVID-19. In multivariable binary logistic regression analysis, only educational status, number of children, and knowledge have remained statistically significant with participants’ level of practice to prevent COVID-19 infection.

Participants who had completed college and above were nearly three times more likely to have an adequate level of practice to prevent COVID-19 (AOR: 3.78; 95% CI: 1.19–11.93) as compared to those who did not attend formal education.

Pregnant women who had less than or equal to three alive children were three times higher the odds of practicing COVID-19 preventive strategies (AOR: 2.89; 95% CI: 1.29–6.45) as compared to women who had more than three alive children.

Once more, study participants who had sound COVID-19 knowledge had eight times higher odds of practicing the preventive measures against COVID-19 (AOR: 8.42; 95% CI: 4.50–15.85) when compared to their counterparts (Table 6).

**Table 6:** Bivariable and multivariable analysis of factors affecting pregnant women level of practice to prevent against COVID-19 infection in Debre Tabor Town Northwest Ethiopia, May 25–June 15, 2020(*N* = 309)

Variables	Level of practice		COR (95%CI)	AOR (95%CI)	*P-value*
	Good N (%)	Poor N (%)			
Educational status					
No formal education	6 (14.6)	36 (85.4)	1	1	
Primary	12 (22.2)	42 (77.8)	**1.667 (1.567–4.897)**	0.748 (0.192–2.912)	0.675
Secondary	13 (22.4)	45 (77.6)	1.685 (0.582–4.885)	0.665 (0.179–2.48)	0.544
College and above	116 (74.4)	40 (25.6)	**6.917 (6.624–23.20)**	**3.783 (1.198–11.938)**	0.023*
Husband educational status					
No formal education	6 (24.0)	19 (76.0)	1	1	
Primary	14 (46.7)	16 (53.3)	2.771 (0.864–8.882)	2.076 (0.351–12.281)	0.421
Secondary	33 (42.3)	45 (57.7)	2.322 (0.836–6.452)	2.404 (0.523–11.046)	0.259
College and above	88 (52.1)	81 (47.9)	**3.44 (1.309–9.041)**	1.115 (0.265–4.688)	0.882
Husband occupation					
Civil servant	91 (52.6)	82 (47.4)	**8.878 (1.087–22.51)**	0.633 (0.45–8.994)	0.736
Private business	41 (43.2)	54 (56.8)	6.074 (0.73–30.50)	0.547 (0.035–8.471)	0.66
Employed at private sector	8 (32.0)	17 (68.0)	3.765 (0.40–22.44)	0.886 (0.051–15.499)	0.934
Daily laborer	5 (50.0)	5 (50.0)	1	1	
Number of children					
≤ 3 children	124 (54.9)	102 (45.1)	**3.171 (1.834–5.483)**	**2.894 (1.298–6.454)**	0.009*
> 3children	23((27.7)	60 (72.3)	1	1	
Condition of pregnancy					
Unwanted	8 (38.1)	13 (61.9)	1	1	
Wanted	132 (52.2)	121 (47.8)	1.773 (0.71–4.424)	1.186 (0.213–6.596)	0.845
Mistimed	7 (20)	28 (80)	0.406 (0.121–1.361)	1.285 (0.156–10.588)	0.816
ANC in current pregnancy					
Yes	127 (53.1)	112 (46.9)	**2.835 (1.591–5.05)**	0.993 (0.381–2.59)	0.989
No	20 (28.6)	50 (71.4)	1	1	
Knowledge level					
Good	134 (78.8)	36 (21.2)	**11 (8.291–23.157)**	**8.421 (4.502–15.85)**	0.000*
Poor	13 (9.4)	126 (90.6)	1	1	

*COR* Crude Odds Ratio, *AOR* Adjusted Odds Ratio

* = *P-value* < 0.05 considered as statistically significant

## Discussion

In this study, the entire participants have been heard about COVID-19. This finding is in agreement with a study done in Bangladesh [21]. Mass media which accounts for 76% was the most likely source of information. Likewise, a study done in Kenya showed that mass media was the main source of information about COVID-19 [22].

According to our study findings, fever and dry cough were the two primary COVID-19 symptoms known by the participants. This finding consistent with a study conducted in Egypt [23]. A systematic review and meta-analysis did by Jafari M. et al. also noticed that fever and cough were the most commonly observed clinical symptoms [14].

The overall knowledge of participants about COVID-19 was 48.6% which was higher than a study done in Egypt (16.39) [23]. This might be due to variations in the educational status of respondents since nearly 60% of the participants attended college and above while 52% of participants attended college and above in the comparative study. Study setting differences may also another reason; since our study was entirely urban setting with high mass media and social media exposure whereas, in Egypt, 20.8% of the participants were rural in residency.

However, it was significantly lower than the finding from Iran (90) [24], Pakistan (93.2%) [25], China 90% [26], and Tanzania (84.4%) [27]. The discrepancy may be due to differences in socio-demographic characteristics, study setting, study participants, and healthcare system of the countries to create awareness regarding the infection.

This study showed that frequent handwashing with water and soap (87.7%), use of face masks in public (77%), and covering mouths and noses when coughing or sneezing (77%) were commonly practiced preventive measures. This finding was supported by a study conducted in Saud Arabia [28].

Based on our study findings, 47.6% of the respondents had a good level of practice to prevent COVID-19. The finding was higher than a study conducted in Nigeria (30.3%) [29]. The possible explanation might be the differences in the level of education of the respondents; only 28.5% had a tertiary level of education while 59.4% of our study respondents had completed college and above. But our study finding was lower than a study done in Pakistan (88.7%) [25]. The disparity may be attributed to variation in study participants since our participants were only pregnant women, while the participants in Pakistan were healthcare professionals who were the front line for the infection.

The result of this study revealed that being in the age category of 15–24 years had an increased level of knowledge towards COVID-19 than the age greater than or equal to 35 years. This finding is not consistent with a study done in China [26], but, it is in line with a study done in Egypt [23]. Participants in this age category (15–24 years) might have easy access to information about the pandemic via mass media and social media since they are highly social media users. Besides, younger women may have higher educational attainment than older ones, which positively affects, their knowledge.

In this study, the odds of having a good knowledge of COVID-19 were higher among women who had attended college and above than those who had no formal education. The finding was supported by a study conducted in Iran [24], China [26], and Egypt [23]. The reason for this might be that educated women can access pertinent information through different mass media and social media including the internet, Facebook, and telegram. They are also more likely to comprehend the information they obtained.

Participants who were civil servants by occupation had a good knowledge index. This finding was supported by a study conducted in China [26]. The possible explanation might be due to employed women may have higher education attainment which influences their knowledge level positively. Similarly, participants who had private business also had good COVID-19 knowledge. These segments of the participants might have linkage and communication with different individuals which positively affect the awareness regarding the outbreak.

Women with wanted pregnancy had good COVID-19 knowledge. As far as the investigator’s knowledge, this is the only study that revealed the condition of pregnancy as a determinant factor for COVID-19 knowledge. The probable reason might be due to women with wanted and planned pregnancies might have an increased healthcare-seeking behavior as early as possible including antenatal care follow-up and gained information from health care providers about the diseases. Besides, unwanted pregnancy may have a psychological impact that negatively affects the whole health of the individual.

Pregnant women who had ANC follow-up in their current pregnancy had a good level of COVID-19 knowledge. To the best of our knowledge, this is also the first finding. The possible explanation may be in addition to routine obstetric care pregnant women who had ANC follow-up can get information about COVID-19 and counsel regarding the contagiously of the pandemic, its multidimensional consequences, and possible prevention methods by the health care provider.

According to the current study finding, women who attended college and above had a good level of practice to prevent the outbreak. This was in agreement with a study finding from Iran [24] and Nigeria [29]. This might be due to educated women can access information easily and discuss more sensitive issues openly and freely since they become closer and familiarized with each other. Also, women with some basic level of education had better understand the complications and consequences associated with not using preventive measures to prevent COVID-19 infection.

Once more, women having less than or equal to three children had a good level of practice. A study from Nigeria also revealed that grand multiparous women (having 5 or above children) had poor practice [29]. The possible explanation might be due as the number of children increase the family size may become crowded which makes it difficult to maintain the recommended distance. Besides, an increased number of children may negatively affect the economy of the family thus less affordable for some preventive measures like soap, alcohol-based hand sanitizer, and face masks that are used to prevent the spread of COVID-19 from person to person.

Lastly, pregnant women who had good COVID-19 knowledge had also good practice to prevent it. A study from China also consistent with this study finding [26]. This might be due to an in-depth knowledge of COVID-19 may increase women’s understanding and awareness of the burden and consequence of the COVID-19 pandemic, and thus, helpful for maintaining safe practices to prevent the infection.

### Limitation

As a limitation due to scarcity of specific kinds of study literature on the participants (pregnant women) the researchers try to discuss with other related studies. This may limit the generalizability of the result. Social desirability and selection bias may also affect the result by preventing the participants from giving truthful information.

## Conclusions

In conclusion, most participants had insufficient knowledge and inadequate practice to prevent COVID-19. Knowledge of participants in the COVID-19 outbreak was significantly associated with age (15–24) years, educational status, occupational status, pregnancy status, and ANC follow-up. Educational status, number of children, and knowledge of COVID-19 were also significantly associated with the practice of preventing COVID-19. As per finding increasing health education programs regarding the pandemic via different media, coordinated and combined efforts of Ethiopian authorities and all individuals will be needed to battle the multidimensional consequences of the pandemic. It is also recommended that mixed methods research, program evaluations, and longitudinal research efforts be undertaken to explore and address the effect of COVID-19 on pregnancy and pregnancy outcomes.

## Supplementary Information

**Additional file 1.** English version questionnaire.

## Data Availability

The datasets used and/or analyzed during the current study are available from the corresponding author on reasonable request.

## Abbreviations

ANC: Ante-natal care
SARS: Severe acute respiratory syndrome
WHO: World Health Organization
