# Supplementary material for: Knowledge and practice to prevent COVID-19 and its associated factors among pregnant women in Debre Tabor Town Northwest Ethiopia, a community-based cross-sectional study
Source: BMC Pregnancy Childbirth. 2021 May 21;21:397. doi: 10.1186/s12884-021-03877-4 (PMC8139538; doi:10.1186/s12884-021-03877-4)
Supplement: Supplementary file 1 — Additional file 1. English version questionnaire. [file 12884_2021_3877_MOESM1_ESM.docx]

**Part I: Socio-demographic characteristics**

| No | Questions | Categories & responses | Skip to |
| --- | --- | --- | --- |
| 101 | Your age? | --------------------year |  |
| 102 | Ethnicity | 1.Amhara  2.Oromo  3.Tigray  4. Other(specify)-------- |  |
| 103 | Your religion? | 1.Orthodox  2. Protestant  3.Catholic  4. Muslim  5. Other (specify) |  |
| 104 | Your education level | 1. Unable to read and write  2. Read and write  3 Primary (1-8)  4 Secondary (9-12)  5.College and above |  |
| 105 | Your occupation | 1.house wife  2.Civil servant  3. Has private business  4. Employed in private sector  5.Others (specify)------- |  |
| 106 | Marital status | 1.Married  2.Widowed  3.Divorced  4.Single |  |
| 107 | Husband educational level | 1. Unable to read and write  2. Read and write  3 Primary (1-8)  4 Secondary (9-12)  5.College and above |  |
| 108 | Husband occupation | 1. Civil servant  2. Has private business  3.Employed in private sector  4.Daily laborer  5.Others (specify)------- |  |
| **Part II: Reproductive health related variables** | | | |
| No | Questionnaire | Categories & responses | Skip to |
| 201 | Gravidity | ------------ |  |
| 202 | Parity | ---------------- |  |
| 203 | Number of alive children | ------------ |  |
| 204 | Do have ANC follow up | 1.Yes  2.No |  |
| 205 | If yes for Q. 204, how many times? | 1.Once  2.Two times  3.Three times  4.Four times |  |
| 206 | Condition of pregnancy | 1.Wanted  2.Unwanted  3.Misstimed |  |
| 207 | Previous adverse pregnancy outcomes | 1.Yes  2.No |  |
| 208 | If yes for Q.207 types of adverse pregnancy outcomes | 1.Abortion  2.Preterm labor  3. Low birth weights  4.Stillbirths  5. Others (specify)--- |  |
| 209 | Do you have history of chronic disease | 1.Yes  2.No |  |
| 210 | If yes for Q 209 which medical disease, do you had | 1.HPN  2.HIV/AIDS  3.DM  4.Renal problem  5.Others(specify)-------- |  |
| **Part III: knowledge factor questionnaires** | | | |
| No | Questionnaire | Categories & responses | Skip to |
| 301 | Have you ever heard about COVID-19 infection? | 1.Yes  2.No |  |
| 302 | If yes for Q.no 301 what is your source of information?(multiple response can be possible) | 1.Massmedia  2.Health professionals  3.Socialmedia(face book,  Google,Telegram,you tube)  4.Others specify)---------- |  |
| 303 | COVID-19 is viral disease | 1.Yes  2.No |  |
| 304 | Respiratory droplets and close contact are the main transmission route | 1.Yes  2. No |  |
| 305 | How long is the incubation period of COVID-19? | 1.1-14 days  2.3-7 days  3.More than 14 days  4.Don’t know |  |
| 306 | Who are susceptible to COVID-19? | 1.The old and children  2.Peoples are generally susceptible  3.Young adult  4.Peoples with pre-exiting disease  5.Pregnant women  6.Don’t know |  |
| 307 | What are the clinical manifestations of COVID-19(multiple response can be possible) | 1.Fever and  2.Dry cough  3. Headache.  4.Sore throat  5. Runny nose.  6.Difficulty of breathing  7. Don’t know |  |
| 308 | What preventive masseurs do you know | 1.Stay at home  2.Wear face mask  3.Others(specify------) |  |
| 309 | Which group of population has poor prognostic outcome if they are infected with COVID-19? (multiple response can be possible) | 1.Adults  2.Childrens  3.Elders(above 60years old)  4.People with co-existing disease (Asthmatic, Hypertensive, DM, Renal and Cardiac…. disease)  5.Pregnant women’s  6. Abusers (smokers…..)  7.Others(Specify)--------  8.Don’t know |  |
| 310 | Person with COVID-19 can transmit the virus to others without development of sign and symptoms? | 1.Yes  2.No  3.Don’t know |  |
| 311 | Are pregnant women more at risk than others? Can the virus be transmitted to the baby? | 1.Yes  2.No  3.Don’t know |  |
| **Part IV practice related questionnaires on COVID-19 prevention methods** | | | |
| No | questionnaire | Categories & responses | Skip to |
| 401 | Did you have practiced COVID 19 prevention methods | 1.Yes  2.No | If yes go to question 503 |
| 402 | If no why not practice COVID-19 prevention methods | -------- |  |
| 403 | Do you wash your hand with soap and water or rubbing with alcohol based sanitizers | 1.Yes  2.No |  |
| 404 | Did you avoid touching eyes, nose and mouth with unwashed hands? | 1.Yes  2.No |  |
| 405 | Do you cover your mouth and nose during coughing or sneezing | 1.Yes  2.No |  |
| 406 | Do you wear face Mask in public | 1.Yes  2.No |  |
| 407 | Do you stay at home or in door? | 1.Yes  2.No |  |
| 408 | Did you maintain at least 1-meter distance from others when u can’t stay in door? | 1.Yes  2.No |  |
